# Impact of Apartment Tightness on Temperature Variability during a Fire

**DOI:** 10.3390/ijerph17124590

**Published:** 2020-06-26

**Authors:** Jerzy Gałaj, Damian Saleta

**Affiliations:** 1 Institute of Safety Engineering, The Main School of Fire Service, 01-629 Warsaw, Poland; 2 Teaching Department, The Central School of State Fire Service, 42-200 Częstochowa, Poland; damian.saleta@gmail.com

**Keywords:** real-scale fire tests, air tightness of residential apartment, fire environment, temperature variability during a fire, fire in apartment

## Abstract

Along with the thermal modernization process of old residential buildings, there has been a significant increase in the air tightness of apartments, which may contribute to the deterioration of the safety of users and rescue teams in a fire. The main goal of this study was to investigate the impact of the air tightness of an apartment on fire growth and temperature variability. In the work, an experimental method was applied. Two full-scale fire tests were carried out, one in a sealed apartment and the other in unsealed one. The temperature was measured by thirty-two thermocouples. Two thermal imaging and video cameras were also used to evaluate a temperature field as well as flame and smoke height. Based on the analysis, conclusions have been formulated. It is noteworthy that the highest temperatures and significant increase in pressure were obtained in a sealed apartment, but dangerous and critical conditions regarding firefighters’ safety were achieved faster and persisted much longer in an unsealed one.

## 1. Introduction

Due to increasing energy costs and requirements for thermal efficiency, many existing buildings in Poland are currently being improved with respect to the insulation properties of the enclosures as well as their air tightness. This process (often referred to as thermo-modernization) is beneficial in terms of the environmental performance of the residential buildings. However, the sealing of apartments can adversely affect the fire environment, e.g., toxicity. This article is a continuation of the topics discussed in the work [1], where, based on experimental research, it was shown that the sealing apartment results in an increase in the concentration of toxic gases in comparison with an unsealed apartment.

As it results from technical and construction regulations, the state of fire safety of a building is determined by the state of the environment in relation to people and by the state of the structure in relation to transferred loads [2]. The critical state of the fire environment is characterized by specific values of its parameters. They refer to thermal decomposition and combustion products, fire temperature and heat radiation power, oxygen concentration and visibility range. The impact of fire on the building structure is usually of a secondary nature, and its dynamic course mainly affects the internal environment of the building and the environment, e.g., the evacuation capabilities of users and the safety of firefighters [3].

Qualitative changes in the technology of construction and modernization of residential buildings caused that currently used building materials and equipment, in combination with modern building structures, can affect some parameters, e.g., temperature during fire in a sealed room. For example, this is indicated by studies carried out in Sweden described in [4]. The study of the impact of room tightness on fire development was presented in the paper basing on a 20-foot container used in the experiment.

The results of American research show that fires nowadays develop faster, more dynamically, emit more heat, are more toxic and go faster to the combustion controlled by ventilation [5]. This information is also confirmed in Polish literature [6,7]. Interesting real-scale fire experiments were carried out in 2006 in Glasgow, Scotland [8,9,10]. The dynamics of fire development in a 22-storey building were analyzed. Furthermore, the impact of various ventilation systems and the fire on the building structure was determined. Realistic fire development scenarios were divided into three groups. The first test concerned the uncontrolled development of fire, while the second test, controlled by ventilation, concerned rooms adapted for the living room/office. The third test concerned a series of tests to check smoke behavior in the stairwell.

Other interesting studies in real-scale were conducted on October 26th, 2011 in France. Fire tests were carried out in a single bedroom apartment (9 m^2^). Scientists studied, among others, which fire effect occurs first in a few simple scenarios using standard ISO 13571 to carry out tenability assessment [11]. Two series of tests were conducted, the first concerned a fire in a closed room, the second with an open door. In connection with the above, the ability to predict temperatures in a residential apartment is of great importance, especially for the possibility of evacuating their users and the safety of operations carried out by firefighters. For this purpose, a number of models are used to calculate the temperature in the rooms [12].

A comprehensive assessment of the air tightness of residential buildings was carried out in Finland [13]. In 2002–2009, the tightness of 170 single-family houses and 56 apartments was checked. The tests have shown very good levels of tightness thanks to many different combinations of insulation and barrier materials. In contrast, the effect of fire on the increase in pressure and ventilation flows was studied during a series of fire experiments in a typical Finnish apartment from the 1970s in the city of Kurikka. They were held in a flat on the first floor (58.6 m^2^) in a three-story building with a basement. The results obtained indicated a significant increase in pressure within a short period of time after the ignition of the fire [14].

It should be noted that the study of temperature and fire development in closed rooms was carried out in an international environment in the years 2006–2011 as part of the PRISME project, mainly in the nuclear area [15]. Extensive research conducted as part of the PRISME project in 2006–2011 (over 35 full-scale fire tests), concerned, among others, fire development in closed and ventilated large representative rooms for the nuclear area. They showed that in the event of a fire in the fire room, overpressures greater than 2500 Pa may occur in conjunction with a significant change in the operating conditions of the ventilation network [16].

In 2010, the Belgian Ministry of Interior funded a study about the fire hazards possibly associated with Passive Houses in comparison to traditional houses. The University of Mons and ISSeP used zone modelling for investigating how much the characteristics of a passive house such as air tightness, ventilation and thermal insulation could affect the fire development. A significant difference in pressure rise (due to thermal expansion of fumes) was observed because of the air tightness in the passive house. However, due to a lack of validation from experimental data on a large scale, the scientific monitoring committee of the project decided to not mention the potential problem that the forces of pressure could block the occupants during a certain period due to inwards-opening doors [17]. Five full-scale experiments were carried out: two in 2016 by Berthelot and three in 2017 by Piret-Gerard. Another experiment related to the increase in pressure and the heat release rate during fires in passive houses was performed by Caravita on 19 December 2017 for needs of MA thesis at the University of Bologna. The research from 2016 and 2017 was carried out on a specially designed hermetic facility that had the same internal dimension as a 40-foot transport container. The experiments carried out indicate the problems associated with overpressure in passive houses in the event of a fire [18].

The purpose of the research, the results of which are presented in this article, was to determine the impact of air tightness of residential apartments on fire development and selected parameters of the fire environment, such as temperature, the height of the flame and smoke-free space and pressure in the context of user and firefighter’s safety.

## 2. Experimental Setup

**Figure 1:**
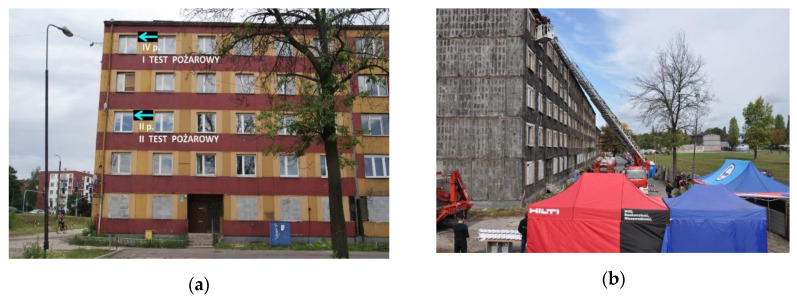
Pictures of a residential building at Pocztowa Str. 15 in Bytom from the north, scheduled for fire tests: (**a**) before the removal of facade panels containing asbestos (July 2012); (**b**) on the day of the experiment (20 September 2012).

The experiments were conducted in a residential five-story building situated in Bytom (Silesian District, Poland), which was intended for demolition. It was built in 1976, made in the technology of a prefabricated reinforced concrete slabs (after removing the facade slabs, it turned out that the building was additionally made of aerated concrete blocks, i.e., gray cellular concrete). The pictures of the building before and after removing the facade panels are presented in Figure 1a,b, respectively.

Two full-scale fire tests were conducted in the building, where two fire scenarios were assumed, for:

1. A sealed apartment No. 19 (poorly ventilated) located on the fourth floor with an area of 36.56 m^2^ (cubic capacity 91.4 m^3^). In the rest of the article, it will be designated by B1 (the first test).
2. An unsealed apartment No. 11 located on the second floor with an area of 37.59 m^2^ (cubic capacity of 93.9 m^3^). In the rest of this article, it will be designated by B2 (the second test).

The room in both apartments, in which the fire source was located, was the same area of 15.41 m^2^.

**Figure 2:**
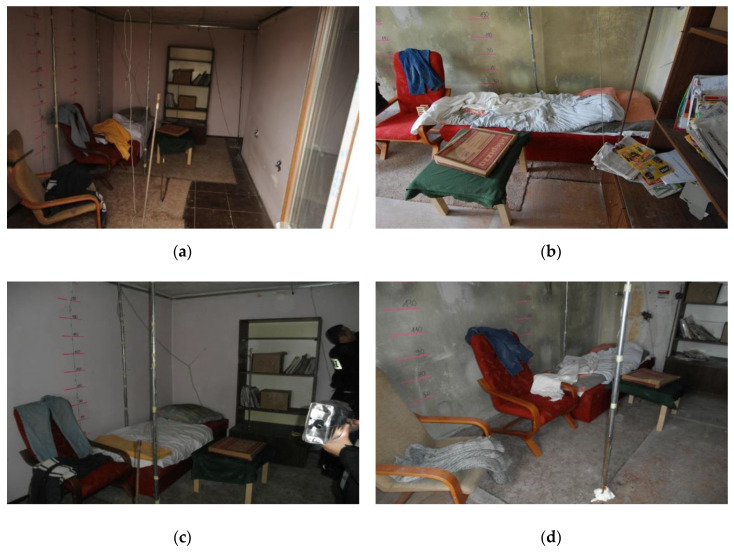
Pictures of rooms where fire tests were carried out: (**a**) sealed; (**b**) unsealed; (**c**) sealed; (**d**) unsealed.

In the sealed apartment, the following configuration of ventilation openings was used: all windows and doors to the apartment were closed, all interior doors were fully open, the vent in the kitchen with dimensions of 14 × 14 cm was open, while such vent were closed in the bathroom. In the unsealed apartment, the same configuration of ventilation openings was used, except for one window from the north whose one wing with dimensions 915 × 1425 cm was tilted 20 cm wide. During both tests, the wind was blowing from the west, but its speed was higher during the fire test in the unsealed apartment (4.5 m/s) than the sealed one (1.5 m/s). In both apartments, the furniture and their settings were identical. They were made to order from the same materials and according to the same pattern (three couches and three armchairs). Each apartment was equipped with the following combustible materials: couch, two armchairs, wall unit, table, blanket, bedding set, tablecloth, pair of trousers, sweater, curtain, and carpet covering 7.5 m^2^. Due to the fact that during the tests the fire was located and no flashover occurred in the entire fire zone, in accordance with the Polish standard PN-B-02852:2001, the actual surface on which the combustible material was located was used to determine the fire load density. It was about 306 MJ/m^2^ for this area (approximately 9 m^2^) and on average 179 MJ/m^2^ for the entire room area with dimensions 520 × 274 × 255 cm (approximately 15 m^2^). Pictures of two rooms before fire tests are shown in Figure 2 (sealed in Figure 2a and unsealed in Figure 2b). Measuring stations for the current registration of results during fire tests were located in rooms directly below the examined apartments, i.e., on the third floor during the first test (B1) and on the first floor during the second test (B2).

The ignition sources and their location (the seat of the armchair next to the couch) during full scale fire tests were identical for both apartments. They consist of two elements:

1. Spruce wood pile made in accordance with British BS 5852 “wood crib 7” (combustion heat 2110 kJ, flame height 345–490 mm, heat flux 25 kW/m^2^, fuel supply indicator 32 g min^−1^, 90% weight loss, time 375 s, burning time 390 s). There was also a blanket and denim jeans on the armchair
2. ROTHENBERGER soldering torch with a disposable container with the MULTIGAS 300 propane/butane gas mixture (35% propane, 65% butane). Technical data: flame temperature up to 1900 °C. Container 600 mL/338g, No.3.5510, designed for all soldering torches and ROTHENBERGER instruments equipped with a connection made according to DIN EN 417. The containers are filled with purified gas. Gas is harmless to the earth’s ozone layer, FCKW-free, TÜV certified

**Figure 3:**
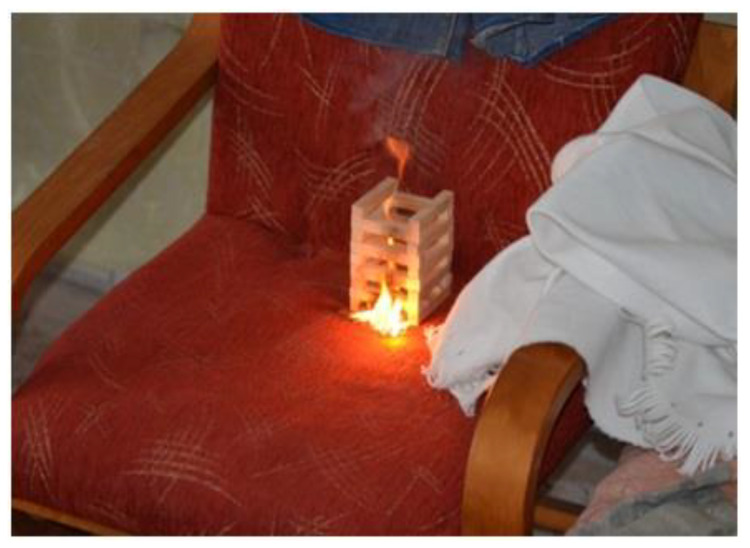
Picture of the place and source of ignition.

The picture of the place and source of ignition are shown in Figure 3.

## 3. Experimental Methods

**Figure 4:**
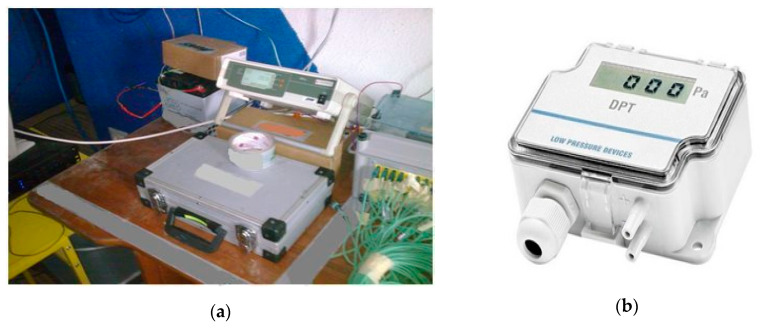
Pictures of: (**a**) analog signal multiplexer MPI-L-16-4-16; (**b**) recorder DPT 01.

**Figure 5:**
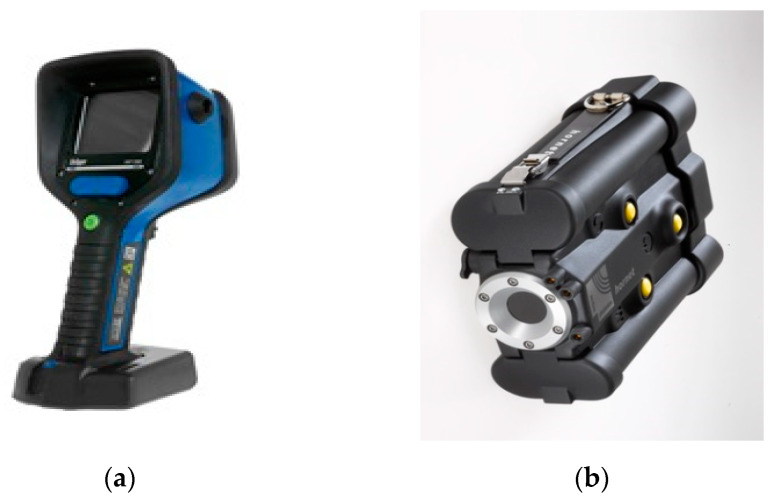
Pictures of two thermal imaging cameras: (**a**) Dräger UCF 7000; (**b**) Hornet 320 B.

The following measuring apparatus was used to measure the distribution and temperature field during the two full-scale fire tests:

1. Thirty-two “K”-type thermocouples (NiCr-NiAl) with a measuring range from −200 to +1200 °C with a mantle sensor. They are mounted on the thermocouple trees marked from t6 to t9, respectively.
2. Three thermocouples type “K” with a wire sensor. They are marked from T1 to T3, respectively. The accuracy of the “K”-type thermocouples was 1.5 °C in the range from −200 to 375 °C and 0.4% in the range from 375 to 1200 °C.
3. MPI-L-16-4-16 analog signal multiplexer with recording function and accuracy in class 1 shown in Figure 4a.
4. Dräger UCF 7000 thermal imaging camera shown in Figure 5a. Temperature measurement in the range from −40 to 1000 °C. Resolution 160 × 120 pixels. Thermal sensitivity 0.035 °C. Tightness class IP 67 [19].
5. Hornet 320 B thermal imaging camera shown in Figure 5b. Temperature measurement in the range from −20 to 590 °C. Spectrum range 7–14 μm. Resolution 320 × 240 pixels. Thermal sensitivity less than 16 mK at f/1.6. Tightness class IP 67 [20].
6. DPT 01 recorder with LCD display shown in Figure 4b. The technical data of it are as follows: 4.20 mA current output and measuring range ± 100 Pa. Measurement accuracy ± 1.5 Pa. The degree of protection IP 54. Equipped with a pressure probe with an aPVC hose [21].

**Figure 6:**
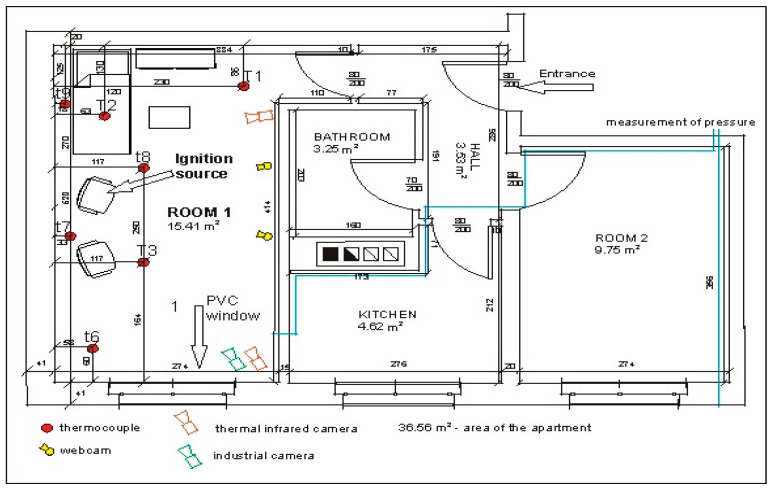
Arrangement of furniture, metal probes, thermocouples tree, thermocouples and cameras in the sealed room.

**Figure 7:**
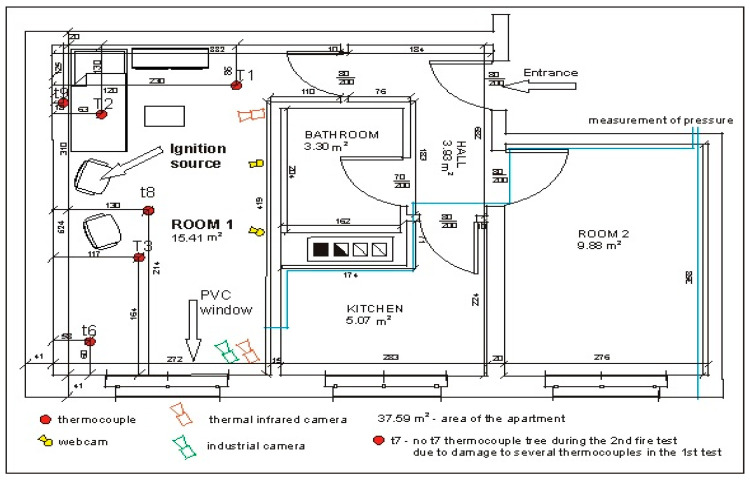
Arrangement of metal probes, thermocouples tree, thermocouples and cameras in the unsealed room.

**Figure 8:**
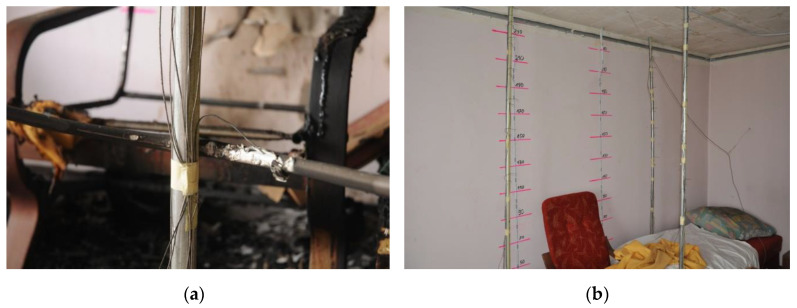
Pictures of: (**a**) a thermocouple T3 placed by the armchair after fire test; (**b**) a scale with height levels on the wall.

In order to measure the temperature inside room 1 during a fire, four thermocouple trees: t6 (near the corner), t7 (between the armchairs near the wall), t8 (between the armchair and the couch in front of them) and t9 (behind the couch near the wall) were installed in it. They were mounted at the following heights measured from the floor in cm: 105, 135, 165, 185, 205, 225, 235 and 245. Due to damage of thermocouples on the t7 tree during the first test (B1), the t7 tree was removed and the position of the t8 tree was slightly changed (see Figure 6 and Figure 7) during the second test (B2). In addition, three single thermocouples (T1, T2 and T3) were placed inside the room (Figure 8a). The thermocouple T1 was mounted at a height of 200 cm near the corridor, T2 at a height of 180 cm over the coach and T3 at a height of 150 cm in front of the armchair. The pressure measuring point was installed near the corner adjacent to the wall with the window at a height of 190 cm. The arrangements of furniture, thermocouples tree, single thermocouples, pressure measuring point (marked by the end of blue line illustrating the wires connecting this point with the recorder) and cameras in the room for sealed and unsealed apartments are shown in Figure 6 and Figure 7, respectively.

Table 1 lists the thermocouple designations on four trees t6, t7, t8 and t9 located inside the room.

The simultaneous recording of temperature in thirty-five (sealed apartment) or twenty-seven (unsealed apartment) measuring tracks took place every 8 s.

The research procedure consisted of the following activities carried out in the listed in order:

1. Preparation of the test apparatus for fire tests:
  - Checking and testing of a computer program for recording and reading temperatures
  - Connection, calibration, and checking the operation of measuring sensors, among others thermocouples, recorders, infrared cameras, smoke detectors or fire alarm system
  - Measurement of initial ambient conditions (wind speed, pressure differences between the building and the environment, and between the staircase and the dwelling, indoor and outdoor air temperature)
  - Preliminary flammability test of the wooden pile made for the requirements of Wood Crib 7 according to the British BS 5852 standard to confirm the height of the flame (in the range 34.5–49 cm) and the time of its complete burning (approximately 6.5 min)
2. Setting an 8-s time step when taking measurements of temperature.
3. Setting fire to the wooden stack placed on the armchair and simultaneously starting the recording of measurements. The possibility of the emergency termination of tests is provided in the event of a situation threatening the safety of the persons carrying out the measurements and who are in the room located under the fire.
4. Completion of the test in a sealed apartment 30 min after ignition and switching off part of the measuring apparatus. The measurement of the temperatures at T1, T2, and T3 continued for 20 min from the end of the test and planned intervention of firefighters (the place of fire was extinguished and a window in the room was opened to smoke the apartment).
5. Firemen putting out the fire and opening a window in the room to smoke the apartment.
6. Checking the building’s safety by a firefighter in charge of Rescue Activities and diagnostics of building structural damage to the building by the District Building Supervision Inspector.
7. Carrying out the second test in the unsealed apartment according to the procedure described in points 1–6.

## 4. Experimental Results

Air tightness measurements for a sealed (B1) and unsealed (B2) apartments according to standard PN-EN ISO 9972: 2015-10 (Blower Door Test) were conducted by ADM Thermo Company, 43-180 Orzesze, Poland. The obtained results are shown in Table 2. Comparing the values given in the table for a sealed room with the recommendations in force in various countries of our climate zone, which can be found, e.g., in [22], it can be stated that the measured air exchange (5.7 m^3^/(h·m^2^)) in most cases does not exceed the maximum recommended level (e.g., 7.8 m^3^/(h·m^2^) in Germany or 10 m^3^/(h·m^2^) in Great Britain).

**Figure 9:**
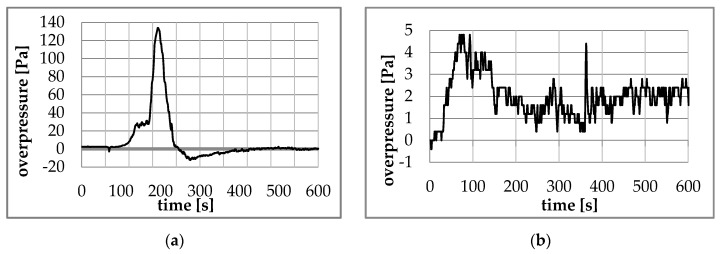
Overpressure curves obtained during test in: (**a**) sealed apartment (B1); (**b**) unsealed apartment (B2).

The pressure distributions measured by recorder DPT 01 for the sealed (B1) and unsealed apartments (B2) are shown in Figure 9a,b, respectively. The ambient pressure was on the day of testing p = 987 hPa. Before testing, the overpressure in the fire-covered room against atmospheric pressure and the pressure difference between the fire zone and the staircase were checked. Both measurements indicated level “0 Pa”. According to measurement data, typical staircase airflow resistance per single floor is in the range 3–5 Pa for the class B system of EN 12101-6 European Standard.

The maximum and minimum pressure values and the time when they were obtained during the B1 and B2 tests are presented in Table 3.

Based on observations and images obtained from thermal imaging cameras and video, the descriptions of fire growth separately for sealed and unsealed apartments are included in Table 4 and Table 5, respectively (the times are counted from ignition).

**Figure 10:**
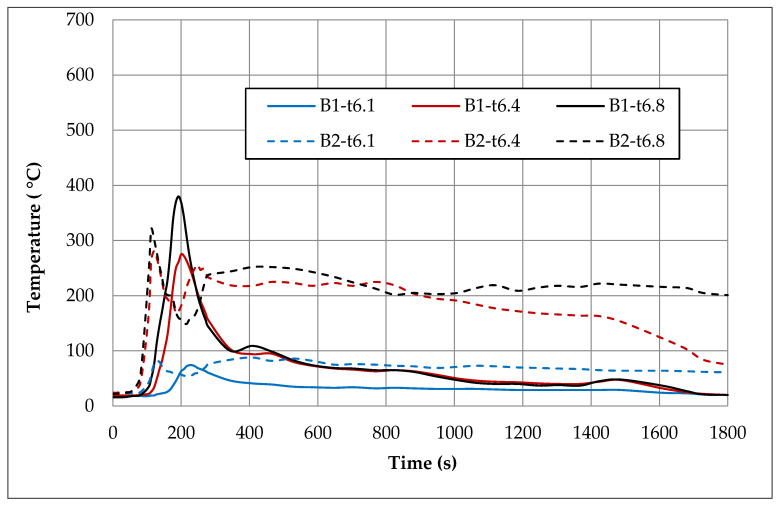
Temperatures measured by thermocouples t6.1, t6.4 and t6.8 during two fire tests B1 and B2.

**Figure 11:**
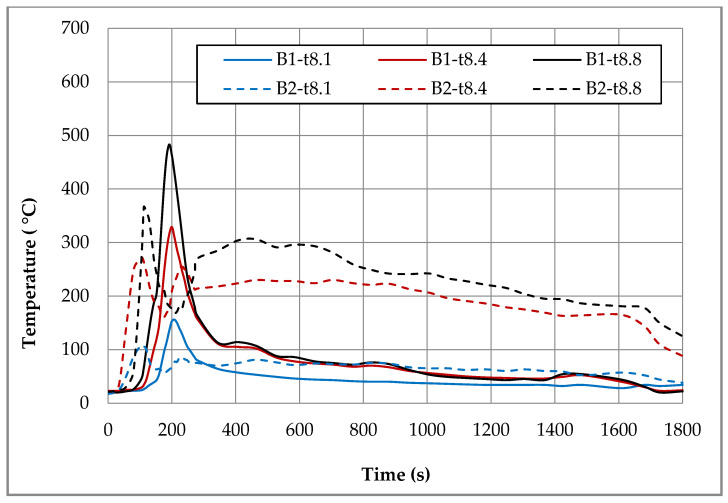
Temperatures measured by thermocouples t8.1, t8.4 and t8.8 during two fire tests B1 and B2.

**Figure 12:**
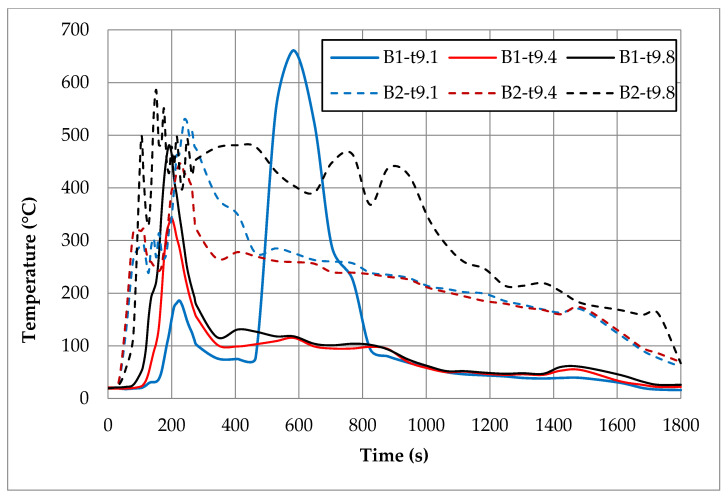
Temperatures measured by thermocouples t9.1, t9.4 and t9.8 during two fire tests B1 and B2.

**Figure 13:**
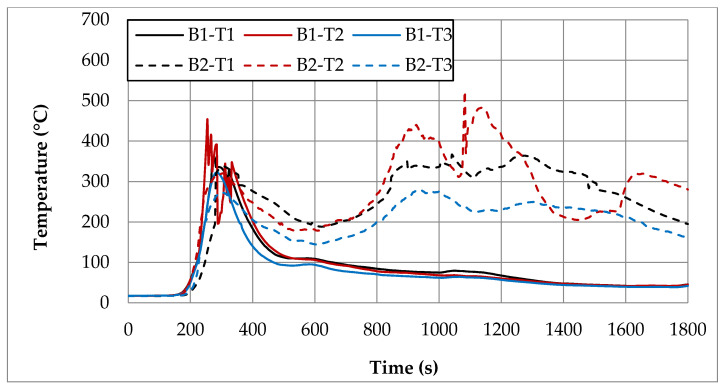
Temperatures measured by thermocouples T1, T2 and T3 during two fire tests B1 and B2.

**Figure 14:**
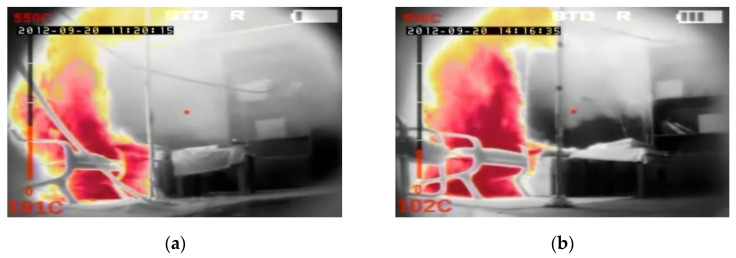
Frames from the movie recorded with thermal imaging camera at 180 s of the fire in the: (**a**) sealed apartment; (**b**) unsealed apartment.

**Figure 15:**
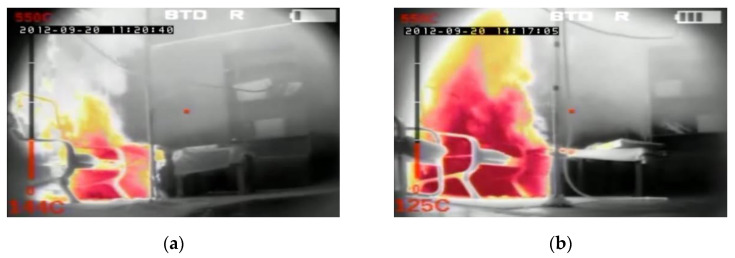
Frames from the movie recorded with thermal imaging camera at 210 s of the fire in the: (**a**) sealed apartment; (**b**) unsealed apartment.

**Figure 16:**
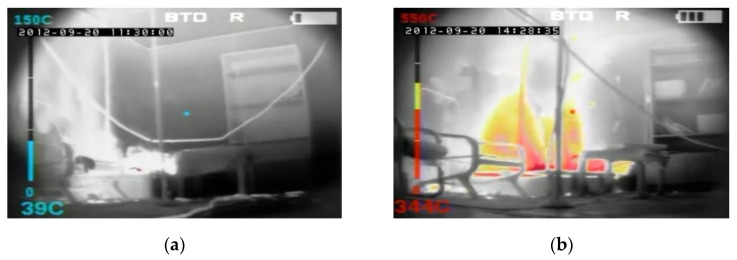
Frames from the movie recorded with a thermal imaging camera at 900 s of the fire in the: (**a**) sealed apartment; (**b**) unsealed apartment.

In order to compare temperatures measured at different heights and in different places of the room during the first fire test (B1) in a sealed apartment and the second fire test (B2) in an unsealed one, temperature changes over time are shown in Figure 10, Figure 11, Figure 12 and Figure 13. Temperature measured by thermocouples on trees t6, t8 and t9 (the measuring path of the t7 thermocouple tree was damaged during the second test) are shown at three selected heights: 105 cm (level above the head of a leaning man), 185 cm (level above the head of a standing or walking man) and 245 cm (ceiling zone). Figure 13 shows the temperature measured by thermocouples T1, T2 and T3. The solid line shows the curve obtained during the first fire test (B1) and the dashed line the curve obtained during the second fire test (B2). To be able to analyze the characteristic quantities associated with the measured temperatures, Table 6, Table 7, Table 8 and Table 9 summarize the maximum and minimum temperatures and the times to reach them. To illustrate the fire history during performed tests frames from the movie recorded with thermal imaging camera for a few selected times are shown in Figure 14, Figure 15 and Figure 16. To assess the temperature values measured during tests B1 and B2 for a critical value of 60 °C at an altitude of 1.85 m adopted in accordance with [23], the times of exceeding it during either temperature increase (time 1) or decrease (time 2) are given in Table 10 (times are counted from the moment of ignition). The values of temperature and height of the smoke layer for selected times of fire during two analyzed tests B1 and B2 are collected in Table 11. They were estimated using a scale applied on the wall (see Figure 8b), thermograms prepared by a thermal imaging camera and recordings from a VHS camera placed in the hallway.

## 5. Discussion

The analysis based on the results presented in Chapter 3 can be found in this section. After analyzing the results of the tightness of the residential apartment, it was found that, in the sealed apartment (B1), a lower number of air exchanges of the entire volume of the apartment in one hour was 5.8 h^−1^, with a difference in internal and external pressure of 50 Pa. In the unsealed apartment the n_50_ value was 11.6 h^−1^. These results have an impact on the permeability and air flow as well as the leakage stream in both analyzed apartments. According to (Journal of Laws of 2017, item 2285), air permeability for windows and balcony doors in low, medium-high and high buildings should be at a pressure of 100 Pa, not more than 2.25 m^3^/(m·h) in relation to up to the contact line length or 9 m^3^/(m·h^2^) in relation to the surface area. In the case of a new window installed in the sealed apartment, the air permeability was 5.7 m^3^/(h·m^2^), while in the unsealed one this value exceeded 9 m^3^/(m·h^2^) and amounted to 11.3 m^3^/(m·h^2^). Research using the Blower Door device showed that the unsealed apartment had lower air tightness (higher unit leakage flow), and the V_50_ air flow was twice as large as in the sealed one. According to the regulation, the number of air changes per hour for buildings with gravitational ventilation should be n_50_ ≤ 3 h^−1^. Therefore, after performing a leak test of both residential apartments by the Blower Door method, it was decided to open one window sash during the second fire test (B2). The reason for this was, among others, the failure of the sealed apartment to meet the assumed leakage test at the level of n_50_ ≤ 3 h^−1^.

During the test in a sealed room (B1), after approximately 120 s after ignition, the pressure in the room began to increase, reaching after 196 s the maximum overpressure value of 134 Pa. The pressure then dropped sharply to reach atmospheric pressure after approximately 240 s. From this moment, a slight increase in underpressure can be observed in the room, which after a time of about 276 s reaches its maximum value of −12.4 Pa. From this moment, the pressure gradually increases to approach the level of atmospheric pressure after about 400 s.

During the test in the unsealed apartment (B2), the overpressure began to increase almost from the very beginning to reach a maximum value of 4.8 Pa after approximately 69 s (about 130 Pa less than in the case of the sealed apartment). At the moment, it decreased slightly. Strong oscillations of overpressure between values of 0.4 and 2.8 Pa can be observed after approximately 150 s (not including a single peak of 4.4 Pa at the 363 s of the test).

The pressure measurement results obtained during the experiments were a big surprise due to the fact that the apartments were not completed in accordance with the number of air changes per hour (n50 ≤ 3 h^−1^) assumed in technical conditions for buildings with gravity ventilation [2].

Due to large differences in the development of the fire during both tests, the analysis of the results obtained was divided into three stages. The first of them analyzed the fire in a sealed apartment, the second in the unsealed one and the third compared the results of both tests B1 and B2.

### 5.1. Fire in the Sealed Apartment (B1)

Two armchairs and the top layer of the couch, which was made from polyurethane foam, were burned out during the B1 test in a sealed apartment. Due to the lack of a sufficiently high temperature, flashover phenomena were not observed. Because of insufficient oxygen, the quenching phase began after just 200 s of fire. Based on the image from the Hornet thermal imaging camera, it can be stated that after only about 20 min the fire was limited to small individual flames appearing on the surface of the couch. The highest intensity of the developed fire was observed after about 170 s of its duration, when the flames reached the ceiling and their length along it was about 120 cm (see Table 11). After about 180 s, the fire passed from the fuel-controlled stage to the ventilation-controlled stage. Moreover, due to the release of a large amount of smoke (insufficient oxygen leads to incomplete combustion), the level of the smoke layer already after approximately 180 s reached the level of 34 cm (calculated from the floor), which then increased, remaining until the end of the test at a height of 70–90 cm. This situation is very dangerous from the point of view of evacuation due to moving in an environment with high toxicity and low visibility range. Based on the temperature characteristics shown in the Figure 10, Figure 11, Figure 12 and Figure 13 and the characteristic values summarized in Table 6, Table 7, Table 8 and Table 9 regarding the B1 test in a sealed apartment, it can be concluded that they were of the same nature at all measured points. It consisted in the fact that after about 100 s from the moment of ignition (which the thermocouple was located lower the later) the temperature began to increase rapidly, reaching its maximum after about 200 s of the fire. The exception here was the local increase in temperature to over 600 °C after about 600 s from the moment of ignition measured by the thermocouple t9.1 caused by the flame coming from the burning couch. Therefore, it was not included in the further analysis. The highest maximum values of around 480 °C were measured in the ceiling zone (245 cm) by t8.8 and t9.8 thermocouples mounted near the ignition source (see Figure 6). The maximum temperature measured by thermocouple t6.8 placed at the same height in the corner of the room was about 100 °C lower. As expected, lower maximum temperatures were obtained at lower altitudes. For example, at a height of 185 cm, these differences ranged from approximately 100 °C (for the t6 tree) to approximately 160 °C (for the t8 tree) and at a height of 105 cm from approximately 300 °C (for the t6 tree) to approximately 330 °C (for the t8 tree). Among the single thermocouples T1, T2 and T3, the highest temperature exceeding 450 °C was indicated by the thermocouple T2 placed above the couch and the lowest equal to approximately 320 °C by thermocouple T3 placed opposite the chair. These values were reached about 70 s later compared to the maxima on the other thermocouples. This is due to the movement of the combustion zone from the armchairs to the couch. In addition, between 270 s and 330 s on the T2 thermocouple, strong and rapid temperature fluctuations can be observed (not found on any other thermocouple) in the range from 200 to 450 °C related to the intense burning of polyurethane foam on the couch mattress. After time when maximum was reached, due to the insufficient amount of oxygen, the fire entered the quenching phase, which resulted in a gradual decrease in temperature. After approximately 400 s from ignition, a smoke height was more than 180 cm and a value of temperature was about 100 °C (at lower altitudes it was several dozen degrees lower). To a level equal to 60 °C accepted as safe for humans at this altitude, the temperature decreased after approximately 1000 s from the moment of ignition.

### 5.2. Fire in the Unsealed Apartment (B2)

During the B2 test in an unsealed apartment, two armchairs and the couch were completely burned out. Because of a too low fire load density, no flashover phenomena were observed. Starting from 50 s, a systematic increase in flame begins, which after 90 s reached a height of 110 cm (calculated from the floor). After about 120 s, the convection column quickly reached the ceiling. After 150 s, the flame spread to the couch. After about 180 s, its rapid spread throughout the couch was recorded, with the flame height reaching ceiling, breaking down along it for a length of about 80 cm. The smoke layer then reached the level of 130 cm above the floor. In the range of 210–300 s, the smoke layer was the lowest equal to approximately 90 cm. This situation at this time practically prevents the evacuation process. Later, it began to gradually increase, reaching a height of approximately 230 cm after 720 s. Combustion intensity dropped after about 300 s, which affected the temperature and flame height drop. Due to the transition of the couch-burning process, which is a significant fire load, to the developed phase, starting from 600 s, the intensity of combustion begins to increase again, reaching its maximum in the range of 900–1200 s. This resulted in a significant increase in temperature and flame height. Practically until the end of the test, which lasted 30 min, the fire was not extinguished, as evidenced by the relatively high temperature and current flame indicated by the thermal imaging camera. Due to the sufficient amount of oxygen, fire is fuel-controlled during the whole test.

During the B2 test in the unsealed room, approximately 70 s after ignition, a fast temperature rise to the maximum begins. The time to reach this temperature depends on the location of the measuring point and the shortest equal to 104 s is for thermocouples t8.4 (185 cm) and t8.1 (105 cm), which are the closest to the source of the fire. For thermocouples mounted at the same heights on the t6 thermocouple tree (in the corner), these times are slightly longer and amount to about 128 s. The highest maximum temperature values at all analyzed levels were measured by thermocouples mounted on a t9 tree located by the wall behind the couch. At a height of 245 cm, it reached the value of 585 °C, while on other thermocouples mounted at the same height it did not exceed 400 °C (366 °C on t8.8 and 320 °C on t6.8). Temperature curves are irregular, consisting in the fact that after reaching the first maximum, the temperature drops momentarily and then increases again to a value that is lower than the previous one in the case of thermocouples t6 and t8, whereas, in the case of the t9 tree and individual thermocouples, T1–T3 is higher. On the t9 tree closest to the couch in the time interval of 100–250 s, significant and relatively fast temperature fluctuations can be observed, which at a height of 245 cm reach up to 250 cm. When it comes to single thermocouples, the highest temperature of 521 °C was recorded on the thermocouple T2 located above the couch. It took place in the range of 900–1100 s, when its combustion was the most intense. At the same time, maximum temperature values were recorded on thermocouples T1 and T3. Until the end of the test, the temperatures they measured did not fall below 150 °C. A high temperature of about 200 °C was also maintained in the ceiling zone in the corner of the room (thermocouple t6.8).

### 5.3. Comparison of both Tests B1 and B2

To compare the two fire tests in the sealed and unsealed apartment, several criteria have been adopted that both characterize the fire itself and affect the degree of danger to people in the apartment. These are: the duration of the fire and its intensity, the height of the flame and its changes in time, the location of the smoke zone, the nature of the temperature changes and its maximum values in the ceiling zone and at the height over a man’s head as well as its value in final phase of the test.

During both tests, the fire was located and did not ignite in the whole fire zone. The fire load density at which the tested materials were collected (9 m^2^ floor area) was 306.4 MJ/m^2^. In contrast, the fire load density for the total floor area was 178.9 MJ/m^2^ for 15.41 m^2^ of the fire room area.

As expected, due to the sufficient amount of oxygen, the fire in the unsealed apartment (B2) was much more intense than in the sealed one (B1). In this case, it was controlled by the fuel throughout the test, while in the sealed apartment after about 180 s it went into the phase controlled by ventilation. Higher intensity affected the flame size. In the case of the sealed apartment, it reached the ceiling only at the beginning of the test. After about 200 s, it began to gradually decrease. In the final phase, only single flames were visible up to a height of 40 cm. During the test in the unsealed apartment, the flame reached the ceiling in the initial phase of the fire lasting up to about 200 s. Then it fell down and its height varied between 70 cm and 90 cm. After approximately 1100 s the flame from the burning couch reached the ceiling again. After about 1620 s of fire, its length along the ceiling was about the width of the couch. Throughout the test, the smoke layer in the sealed apartment was located much lower than in the unsealed apartment. After about 180 s, it reached the lowest position of 34 cm (calculated from the floor), after which it rose a bit by turning around 70–90 cm. In the unsealed apartment, about 180 s after ignition, it dropped to the level of 130 cm, to continue to remain at a safe level for humans (230–240 cm) until the end of the test.

Due to significant differences in the combustion process resulting primarily from a much larger amount of available oxygen during tests in the unsealed apartment, significantly different temperature characteristics were observed for both analyzed apartment. In the case of the sealed apartment, after reaching the maximum, it first decreases quickly to a level of about 100 °C and then it slows down much slower until the end of the test with a slight fluctuation around 400 s related to the transition of the flame to the couch and the combustion of its top layer. In the case of the unsealed apartment, when the temperature reaches the first maximum, a sharp decrease can be observed, but only to a value that was not lower than 150 °C, and then a further increase associated with the transition to the phase in which the entire couch is burning. This causes a high temperature to remain (at a height of 245 cm it ranges between 200–300 °C) for a long time. Only with the beginning of the phase quenching after a period of about 800–1000 s after ignition (it depends on the location of the measuring point) does it begin to gradually decrease in temperature. Comparing the maximum temperatures recorded in the ceiling zone with each other, it can be stated that the values measured on the trees t8 (between the fireplace with the source of fire and the couch), t6 (in the corner of the room) and the single thermocouple T3 (opposite the armchair) were higher by about 40–80 °C for the sealed apartment. The opposite was true for the t9 tree behind the couch. The temperature measured by thermocouple t9.8 was about 100 °C higher in the unsealed apartment. As for the thermocouple T1 (furthest from the combustion zone), the maximum temperatures were comparable in both examined cases. In turn, the maximum temperature measured by the thermocouple T2 placed above the couch was higher by about 70 °C during the test in the unsealed apartment. It was achieved only in the third phase when burning the couch (after about 1083 s). During the test in a sealed apartment after about 1200 s, the temperature at all analyzed measuring points dropped to a level 60 °C safe for humans. However, during the test in the unsealed apartment, the temperature value was measured at practically all measuring points (except for the lowest located at a height of 105 cm) and exceeded this value.

The uncertainty in determining the parameters of the fire environment resulted not only from the limited accuracy of instruments and measurement methods, but above all from the stochastic nature of the phenomenon that is fire. This is also associated with the fact that it is virtually impossible to provide the same conditions for the development of fire in both apartments differing only in the degree of air tightness. The uncertainty of results was also caused by the difference between the wind speed blowing during the test in sealed (1.5 m/s) and unsealed (4.5 m/s) apartments. Assuming that the windows in both apartments were located on the north side, this could affect the maintaining of higher temperatures in the unsealed apartment for a longer period and hence faster burning of furniture. The influence of cool air and wind on the direction and intensity of fire in the unsealed apartment was also visible on the T3 thermocouple (150 cm from the floor by the chair), because the highest temperature at this point was obtained in the sealed apartment. In addition, a small number of fire tests have an impact on the accuracy of the results obtained. It resulted from technical problems and limited financial and personal resources.

## 6. Conclusions

On the basis of the analysis of results obtained during full-scale fires included in Chapter 5, the following final conclusions have been formulated:

1. Taking into account the intensity of the fire and its duration, which had a significant effect on maintaining a high temperature in the room for a longer time, a greater threat to humans, mainly due to the effects of thermal radiation, is a fire in the unsealed apartment. In the case of the sealed apartment due to the limited amount of oxygen, after about 900 s its intensity decreased so that the height of individual flames above the couch did not exceed 40 cm and the temperature at a height of 185 cm fell below 80 °C (during the whole test the seats and only the upper part of the couch burned out). At the same time, in the sealed apartment, the developed combustion phase of the couch began, during which the height of the flames reached the ceiling and the temperature near the couch practically remained at the level of 200 °C until the end of the test.
2. Considering the location of the smoke layer due to its significantly lower height above the floor (in the initial phase of the fire in a sealed room it even dropped to 34 cm, then increased slightly, but practically remained at a level not higher than 70 cm until the end of the test), due to the toxicity of the products contained in the smoke (especially carbon monoxide emitted during incomplete combustion), a reduction in oxygen concentration and visibility range, a fire in the sealed apartment is a greater threat to humans. Moreover, in this situation, there is a greater probability of a possible backdraft phenomenon, for example, in the case of a supply of oxygen as a result of opening the apartment door by fireman or cracking the glass due to its heating up to a temperature exceeding 300 °C. In the unsealed apartment, the lowest location of the smoke layer in the initial phase of the test was nearly 130 cm, while starting from about 600 s it remained at a safe height for people (more than 200 cm).
3. Due to a much larger amount of oxygen in the unsealed apartment (test B2), measured temperatures were significantly different from that obtained in the test B1 (sealed apartment). In the first case (B1), due to a lack of sufficient oxygen, the fire lasted much for less time and was limited to only burning the armchair and its immediate surroundings. This is demonstrated by the temperature curve containing only one maximum. When it was reached, the temperature continued to decrease until the end of the test. In the case of apartment unsealed, there were a few local temperature maxima. It resulted, among others from the fact that in addition to the armchair, the fire switched to the couch, which resulted in the further phase of the fire (in the range from 900 to 1500 s) again more intense heat release resulting in a re-increase in temperature.
4. The maximum temperature values recorded, except for one case when the t9 thermocouple was in direct flame range, did not exceed 600 °C. The highest temperatures exceeding 450 °C were measured by thermocouples mounted on the t9 tree closest to the original source of fire. In this case, they were higher in the test B2 (apartment unsealed). As for the remaining thermocouple trees, higher maximum temperatures were most often obtained during the test B1 (sealed apartment). The temperature measured at a height of 185 cm (above the head of a standing man of average height) after about 80 s (less than 1.5 min) in the case of the unsealed apartment and 120 s (2 min) in the case of the sealed apartment exceeded the value constituting a threat to health and life human (assumed to be 60 °C). At a height of 105 cm (man in an inclined position), this time was slightly longer and in the worst case it was 90 s (thermocouple t9.1 during test B2) and in the best 200 s (thermocouple t6.1 during test B1).
5. From the point of view of human safety, exceeding the critical value of 60 °C at a height 1.85 m occurred the fastest (after about 85 s) in the case of test B2 (unsealed apartment), and the latest (after about 134 s) in the case of test B1 (sealed apartment). Differences between times depending on the location of the thermocouple tree did not exceed 10 s. A temperature drop to the level of 60 °C in the quenching phase was observed only in the case of the B1 test in a sealed apartment. In the B2 test, they were above the critical value until the end of the measurements. The fastest decrease occurred in the case of the t6 tree placed in the corner of the room and at the latest in the case of the t9 tree located behind the couch.

The temperature curves obtained as a result of full-scale fire tests can be used to estimate the heat release rate (HRR curves) using the known inverse method, e.g., the one given in [24]. In addition, they can be useful when validating either a computer zone or field fire model. The above-mentioned issues will be the subject of another article related to conducted fire experiments.

## Figures and Tables

**Table 1:** Thermocouples markings.

No.	Height Measured from the Floor in cm	Thermocouple Tree			
		t6	t7	t8	t9
1	105	t6.1	t7.1	t8.1	t9.1
2	135	t6.2	t7.2	t8.2	t9.2
3	165	t6.3	t7.3	t8.3	t9.3
4	185	t6.4	t7.4	t8.4	t9.4
5	205	t6.5	t7.5	t8.5	t9.5
6	225	t6.6	t7.6	t8.6	t9.6
7	235	t6.7	t7.7	t8.7	t9.7
8	245	t6.8	t7.8	t8.8	t9.8

**Table 2:** Values of air flow, number of air changes, air permeability and unit leakage stream measured during two analyzed tests B1 and B2.

Parameter	Notation	Unit	Results		Uncertainty
			B1	B2	B1 and B2
Air flow	V_50_	m^3^/h	549	1098	±7%
Number of air changes	n_50_	h^−1^	5.8	11.6	±9%
Air permeability	q_50_	m^3^/(h·m^2^)	5.7	11.3	±9%
Unit leakage stream	w_50_	m^3^/(h·m^2^)	14.5	28.9	±9%

**Table 3:** Maximum and minimum values of pressure obtained during tests B1 and B2.

Parameter	Pressure (Pa)		Time (s)	
	B1	B2	B1	B2
Maximum	134.0	4.8	193	69
Minimum	−12.4	0.4	276	248

**Table 4:** Description of fire growth during test in the sealed apartment.

From (s)	To (s)	Description
0	24	A gas burner was used to initiate a fire in a sealed apartment, with which a pile of spruce wood was placed on a chair near the couch. The ignition time was 5 s.
24	49	The fire growth phase began. There was a moment of heating, thermal decomposition and ignition of materials around the pile (part of the seat and back of the armchair). The flame height was in the range from 34 cm to 49 cm.
49	99	The accelerated thermal decomposition and combustion of the armchair occurred. Flame propagation took place in the direction of convective flow over the surface of the burning armchair. The flame reached a height of 130 cm above the floor.
99	124	The armchair with ignition source burned out. The smoke surface in the ceiling increased. The flame reaches 210 cm above the floor.
124	199	The fire spread to the couch and the temperature increases steadily. In this time period (approximately after 180 s from ignition) the fire passed from the fuel-controlled phase to the ventilation-controlled phase. The smoke layer lowers from the ceiling level to a height of about 150 cm.
199	224	A decrease in combustion intensity was observed. The surface of the convection column of fire has decreased. The smoke increased and the height of the diffusion flame decreased to a level about 150 cm above the floor. The smoke layer remained at a level of about 40 cm from the floor.
224	250	There was a further decrease in the intensity of flame combustion caused an increase in the evolution of toxic combustion products and combustible fire gases. Only part of the couch surface was affected by the fire. A low flame approximately 70 cm high was observed on the couch surface. The smoke layer remained at a level of approximately 40 cm above the floor.
250	275	A systematic decrease in the rate of heat released, reduction in the combustion area and an increase in intensity of the release of incomplete combustion products. The smoke layer was still around 40 cm above the floor.
275	400	The temperature dropped to 85 °C. The smoke layer increased to approximately 50 cm above the floor.
400	1200	The flame was maintained at a height of approximately 70 cm in the central part of the couch and the continuous release of smoke from the burned part of the mattress was observed. The smoke layer did not change and remained at a level of approximately 50 cm above the floor. The average temperature was approximately of 50 °C.
1200	1800	Only single tongues of fire were found on the couch surface at a height of approximately 40 cm above the floor. The temperature displayed on the thermal imager was 34 °C. The smoke layer was approximately of 70 cm above the floor.

**Table 5:** Description of fire growth during test in the unsealed apartment.

From (s)	To (s)	Description
0	30	A gas burner was used to initiate a fire in a sealed apartment, with which a pile of spruce wood was placed on an armchair near the couch. The ignition time was 4 s.
30	60	The initial phase of fire growth, heating and thermal decomposition of the surroundings of the fire source occurred (part of the seat and back of the armchair). The flame height was approximately 50 cm above the floor.
60	90	There was still local combustion. The fire developed more slowly at this stage than during the first test. A smoky layer began to form in the upper part of the room. There was a systematic increase in flame height.
90	120	The flame was approximately at a height of 110 cm above the floor. From this moment faster burning and increase of fire development were observed.
120	150	The rapid growth of the convective column and faster heat transfer generated by the flame (heat radiation flux density), which reached the ceiling were observed. The temperature on the thermal imager increased slightly to 27 °C. A layer of smoke was formed.
150	180	The combustion zone increased. The fire spread to the couch, where rapid heating and thermal decomposition of the entire surface of the material covering the mattress was observed. Inflammation of the carpet between the armchair and the couch was also noticed. In the ceiling zone, the density of the smoke layer increased as a result of the extraction of pyrolysis products running deep into the mattress polyurethane foam.
180	270	The temperature increased rapidly. The rapid spread of flame over the entire surface of the couch was observed. The flame height reached the ceiling and collapsed along the ceiling for a length of approximately 80 cm (couch width). The smoke layer reached up to approximately 130 cm above the floor.
270	720	The flame height lowered to a level of 150 cm above the floor, due to the intake of cold air through the window and heat loss on the ceiling surface (high thermal conductivity). The temperature indicated by the thermal imaging camera dropped slightly to approximately 110 °C, which confirms the decreasing heat flux in the combustion zone. Smoke layer was about 90 cm above the floor.
720	900	A significant increase in the intensity of the fire was noticed. The flame reached a height of 110 cm above the floor. The temperature increased to 97 °C. The smoke layer increased to a level of approximately 230 cm above the floor.
900	1200	The maximum temperature displayed on a thermal imaging camera was 340 °C. The whole area of the couch was covered by flame which reached the height of 170 cm above the floor. The smoke layer was located at a height of 240 cm above the floor
1200	1500	The temperature indicated by the thermal imaging camera dropped to 284 °C. The burning intensity was higher in the front of the couch (in the corner of the room). The height of the flame reached the ceiling level. Low smoke density was observed.
1500	1800	The temperature increased again to 325 °C. The height of diffusion flame reached 170 cm above the floor. In the final phase of the test the flame was 100 cm above floor. The temperature displayed on the thermal imager decreased to 223 °C.

**Table 6:** The characteristic parameters of temperature curves registered by thermocouples t6.1, t6.4 and t6.8 during two fire tests B1 and B2.

Parameters	t6.1		t6.4		t6.8	
	B1	B2	B1	B2	B1	B2
Maximum (°C)	74	88	275	281	380	320
Time to maximum (s)	224	128	200	128	192	112
Minimum (°C)	*	55	*	172	*	149
Time to minimum (s)	*	216	*	192	*	216

* practically no minimum local temperature, because, in the first test, after reaching the maximum, it gradually decreases until the end of the test.

**Table 7:** Characteristic parameters of temperature curves registered by thermocouples t8.1, t8.4 and t8.8 during two fire tests B1 and B2.

Parameters	t8.1		t8.4		t8.8	
	B1	B2	B1	B2	B1	B2
Maximum (°C)	156	108	329	271	483	366
Time to maximum (s)	208	104	200	104	192	112
Minimum (°C)	*	55	*	161	*	171
Time to minimum (s)	*	176	*	176	*	216

* practically no minimum local temperature, because, in the first test, after reaching the maximum, it gradually decreases until the end of the test.

**Table 8:** Characteristic parameters of temperature curves registered by thermocouples t9.1, t9.4 and t9.8 during two fire tests B1 and B2.

Parameters	t9.1		t9.4		t9.8	
	B1	B2	B1	B2	B1	B2
Maximum (°C)	179 (661)	530	343	446	481	585
Time to maximum (s)	232 (584)	240	200	232	192	152
Minimum (°C)	* (75)	239	*	242	*	329
Time to minimum (s)	* (408)	128	*	160	*	128

* practically no minimum local temperature, because, in the first test, after reaching the maximum, it gradually decreases until the end of the test.

**Table 9:** Characteristic parameters of temperature curves registered by thermocouples T1, T2 and T3 during two fire tests B1 and B2.

Parameters	T1		T2		T3	
	B1	B2	B1	B2	B1	B2
Global maximum (°C)	363	366	453	521	323	280
Time to global maximum (s)	276	1041	255	1083	285	933
The first local maximum (°C)	**	337	**	320	**	272
Time to the first local maximum (s)	**	306	**	300	**	300
Local minimum (°C)	*	190	196	178	*	144
Time to local minimum (s)	*	639	288	609	*	609

* practically no minimum local temperature, because, in the first test, after reaching the maximum, it gradually decreases until the end of the test. ** the first local maximum is also global maximum.

**Table 10:** Values of times after which the temperature measured by thermocouples t6.4, t8.4 and t9.4 reached a critical value of 60 °C during its growth and drop.

No. of Thermocouple	B1		B2	
	time 1 (s)	time 2 (s)	time 1 (s)	time 2 (s)
t6.4	134	880	85	* (84 °C)
t8.4	128	944	95	* (67 °C)
t9.4	134	1005	87	* (69 °C)

* during the B2 test (unsealed apartment), the temperature at a height of 1.85 m did not drop below 60 °C by the end of the test (in brackets the value measured at the end of the test is given).

**Table 11:** Values of temperature and height of the smoke layer for selected times of fire during two analyzed tests B1 and B2.

Parameters	Time [s]									
	180		210		360		540		720	
	B1	B2	B1	B2	B1	B2	B1	B2	B1	B2
Temperature (°C)	191	120	144	125	55	98	46	88	38	97
Flame height (cm)	255	255	150	255	90	130	90	90	90	110
Flame length under the ceiling (cm)	120	80	0	0	0	0	0	0	0	0
Smoke height (cm)	34	130	40	165	50	150	70	210	90	230
**Parameters**	**900**		**1080**		**1260**		**1440**		**1620**	
	**B1**	**B2**	**B1**	**B2**	**B1**	**B2**	**B1**	**B2**	**B1**	**B2**
Temperature (°C)	38	344	37	218	34	284	32	320	32	405
Flame height (cm)	70	170	50	130	40	255	40	190	40	255
Flame length under the ceiling (cm)	0	0	0	0	0	0	0	0	0	80
Smoke height (cm)	90	240	90	230	70	240	70	230	70	240
